# *Dlx5*-augmentation in neural crest cells reveals early development and differentiation potential of mouse apical head mesenchyme

**DOI:** 10.1038/s41598-021-81434-x

**Published:** 2021-01-22

**Authors:** Tri H. Vu, Masaki Takechi, Miki Shimizu, Taro Kitazawa, Hiroki Higashiyama, Akiyasu Iwase, Hiroki Kurihara, Sachiko Iseki

**Affiliations:** 1 Section of Molecular Craniofacial Embryology, Graduate School of Medical and Dental Sciences, Tokyo Medical and Dental University (TMDU), 1-5-45 Yushima, Bunkyo-ku, Tokyo, 113-8549 Japan; 2 Department of Physiological Chemistry and Metabolism, Graduate School of Medicine, The University of Tokyo, 7-3-1 Hongo, Bunkyo-ku, Tokyo, 113-0033 Japan

**Keywords:** Bone development, Cartilage development, Differentiation, Embryogenesis, Morphogenesis

## Abstract

Neural crest cells (NCCs) give rise to various tissues including neurons, pigment cells, bone and cartilage in the head. *Distal-less homeobox 5* (*Dlx5*) is involved in both jaw patterning and differentiation of NCC-derivatives. In this study, we investigated the differentiation potential of head mesenchyme by forcing *Dlx5* to be expressed in mouse NCC (*NCC*^*Dlx5*^). In *NCC*^*Dlx5*^ mice, differentiation of dermis and pigment cells were enhanced with ectopic cartilage (ec) and heterotopic bone (hb) in different layers at the cranial vertex. The ec and hb were derived from the early migrating mesenchyme (EMM), the non-skeletogenic cell population located above skeletogenic supraorbital mesenchyme (SOM). The ec developed within *Foxc1*^+^-dura mater with increased PDGFRα signalling, and the hb formed with upregulation of BMP and WNT/β-catenin signallings in *Dermo1*^+^-dermal layer from E11.5. Since dermal cells express *Runx2* and *Msx2* in the control, osteogenic potential in dermal cells seemed to be inhibited by an anti-osteogenic function of *Msx2* in normal context. We propose that, after the non-skeletogenic commitment, the EMM is divided into dermis and meninges by E11.5 in normal development. Two distinct responses of the EMM, chondrogenesis and osteogenesis, to *Dlx5*-augmentation in the *NCC*^*Dlx5*^ strongly support this idea.

## Introduction

Neural crest cells (NCCs) are mesenchymal cells that originate from the dorsal part of neural tube by epithelial-to-mesenchymal transition. NCCs then migrate to different regions of the embryo, where they give rise to various cell types such as bone and cartilage of the skull, sensory neurons, pericytes, melanocytes and smooth muscles^1^. NCCs and paraxial mesodermal cells (MES) cooperatively form the craniofacial structure. NCCs contribute to the rostral craniofacial skeleton including the pharyngeal skeleton while MES give rise to caudal cranium^2–4^. The boundary between NCC and MES in the calvarium corresponds to the coronal suture between the frontal and parietal bones^3–5^.

The NCC is accurately regulated by a complex gene network from the appearance to migration and differentiation^1^. *Distal-less homeobox 5* (*Dlx5*) is expressed early at the neural plate border for establishing the area of NCC delamination, but it does not control NCC specification or migration^1,6^. *Dlx5* is required for patterning and differentiation of the NCC^7^. Expression of *Dlx5* and its co-functional member of the *Dlx* gene family, *Dlx6*, are involved in jaw patterning^8,9^. In jaw development, *Dlx5/6* works downstream of *Endothelin1* (*Edn1*), localized in the mandibular process while *Dlx5/6* are absent in the maxillary process^8–12^. Double knock-out of *Dlx5/6* in mice causes mandible transformation into maxilla-like structure^8^. Reversely, forced *Dlx5* expression in NCCs in mice (*NCC*^*Dlx5*^) induces ectopic *Dlx5* expression in the maxillary process leading to upregulation of mandibular-specific genes and appearance of several phenotypic hallmarks of the mandible in the maxilla region^11^.

*Dlx5* is also expressed in differentiation stages of NCC-derivatives: ganglion of cranial nerves, cartilage and bone^13,14^. In osteoblast differentiation, *Dlx5* is induced by BMP signalling, then *Dlx5* enhances *Runt-related transcription factor 2* (*Runx2*), a master transcriptional regulator for osteogenesis^15–17^. DLX5 directly binds to SP7, a downstream of *Runx2*, to promote osteoblast differentiation^18^. Calvarial osteoblasts isolated from *Dlx5* deleted mice show reduced proliferation and differentiation^19^. *Dlx5* is also expected to induce recruitment of fibroblasts to chondrogenic lineage and chondrocyte maturation in the chick^20,21^. In mice, the calvarium and chondrocranium malformations have been shown to associate with *Dlx5*-downregulation^7,22,23^. Meanwhile, cranial base cartilages derived from NCC are enlarged by *Dlx5*-overexpression, but calvarial bones have not been examined^11^.

In calvarial development, formation of the frontal and parietal bones start with the aggregation of mesenchymal cells in the area of the supraorbital ridge at embryonic day (E) 10.5^3^, referred to as the supraorbital ridge mesenchyme^5,24–26^ or supraorbital mesenchyme (SOM)^27^. The SOM proliferates and differentiates into osteoblasts from E11.5, then intrinsically expands to the apex of the head to form the bone from E13.5^4,5^. Importantly, due to the intimate association and mutual support of cranial bones and the dura mater, the defects in the dura mater affect calvarial bone formation and maintenance^28–31^. Before the SOM begins apical growth, a population of head mesenchyme, termed as early migrating NCC^32^ or early migrating mesenchyme (EMM)^27^, is established above the SOM to contribute to the sutures or soft tissue layers such as the dermis and the meninges^4,25,32^. Transcriptome analysis revealed that the SOM and the EMM exhibit different gene expression profiles by E12.5^33^, and the development of the skull vault is achieved by interactions between the apical (EMM) and basal (SOM) cell populations^28^. Although the EMM is normally non-osteogenic, previous reports demonstrated that the EMM can generate bone in genetic disorders^27,32^.

NCC-specific *Dlx5*-augmentation results in a switch of the jaw identity^11^, but the effect on NCC differentiation potential has not been examined. In this study, we further investigated the effect of *Dlx5*-overexpression in NCCs with special reference to early development and differentiation potential of the EMM.

## Results

### ***Dlx5*** expression and NCC distribution in the ***NCC***^***Dlx5***^

**Figure 1:**
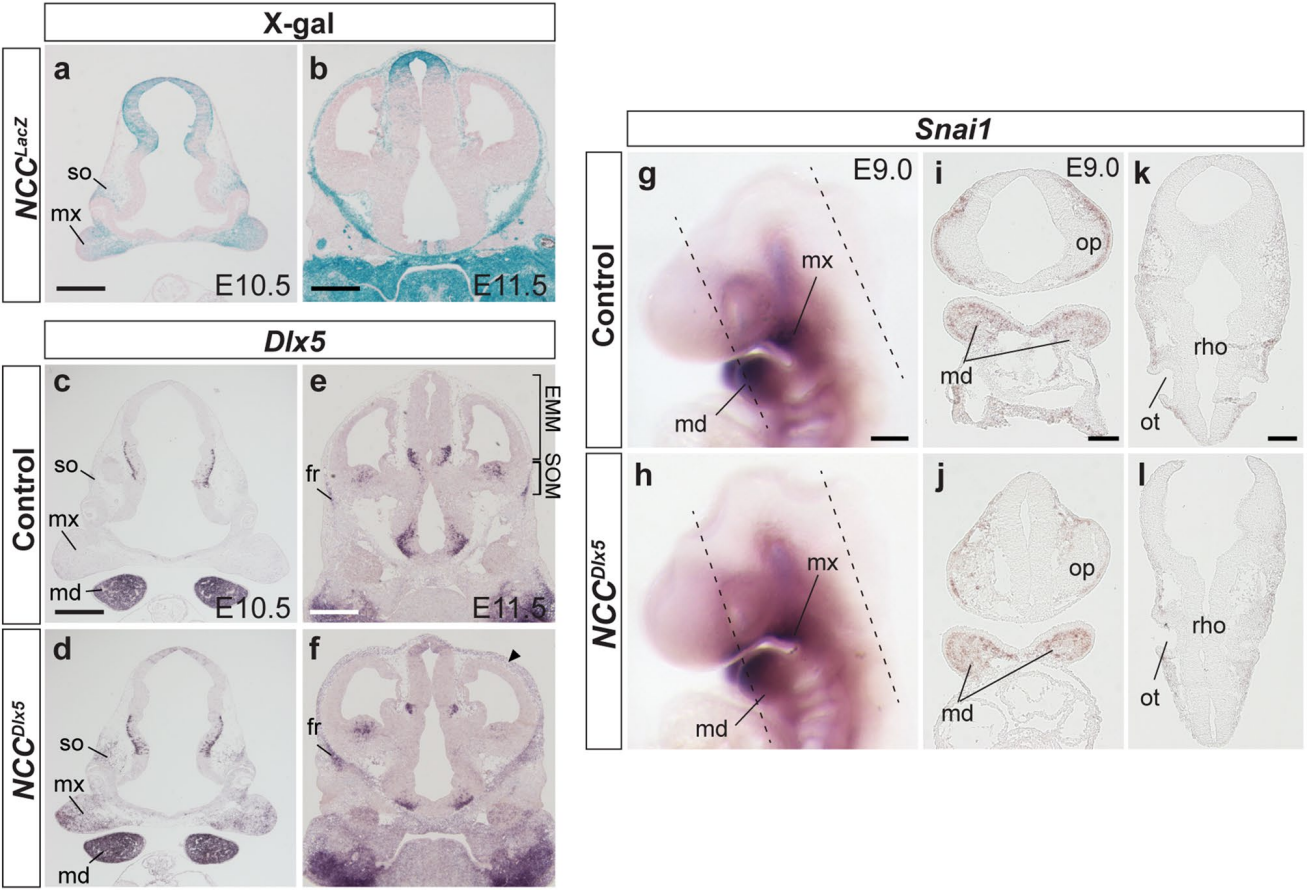
Ubiquitous *Dlx5* expression in NCCs and the effect on the distribution of NCCs in the *NCC*^*Dlx5*^. (**a**, **b**) X-gal staining of *NCC*^*LacZ*^ counterstained by nuclear fast red to visualize NCCs at E10.5 and E11.5. (**c**–**f**) *Dlx5* expression on the frontal section of the control and the *NCC*^*Dlx5*^ at E10.5 (**c**, **d**) and E11.5 (**e**, **f**). In the *NCC*^*Dlx5*^, *Dlx5* is ectopically expressed in maxilla and supraorbital mesenchyme at E10.5 (**d**), and in apical head mesenchyme at E11.5 (**f**, arrowhead). (**g**, **h**) *Snai1* expression, a marker for NCC, by WISH of the control and the *NCC*^*Dlx5*^ at E9.0. (**i**–**l**) *Snai1* expression by ISH on frontal sections of the control and the *NCC*^*Dlx5*^ at E9.0, at planes corresponding to hatched lines in (**g**, **h**), showing the forebrain, the mandibular process (**i**, **j**), and the dorsal area (**k**, **l**). fr, frontal bone; md, mandibular process; mx, maxillary process; so, supraorbital ridge; EMM, early migrating mesenchyme; SOM, supraorbital mesenchyme; op, optic vesicle; ot, otic vesicle, rho, rhombencephalon. Scale bars; 500 μm (**a**, **b**, **c**, **e**), 200 μm (**g**), and 100 μm (**i**, **k**).

NCC-specific forced expression of *Dlx5* was confirmed in *Wnt1-Cre;R26R*^*CAG-flox-Dlx5/*+^ (hereafter *NCC*^*Dlx5*^) mice at E9.5 with ectopic *Dlx5* expression in the pharyngeal arch^11^. We further examined the expression of *Dlx5* in later stages by comparison with X-gal staining of *Wnt-Cre;R26R*^*lacZ/*+^ (*NCC*^*LacZ*^) (n = 3) to demonstrate the NCC distribution. NCCs of wild-type located at the maxillary process and supraorbital ridge at E10.5, but hardly detected in the surrounding of the brain at the vertex (Fig. 1a). At E11.5, NCCs made up the mandibular and maxillary processes, also the head mesenchyme surrounding the brain (Fig. 1b). Endogenous *Dlx5* expression in head mesenchyme at E10.5 was found only in the mandibular process, whilst in the *NCC*^*Dlx5*^*, Dlx5* was additionally expressed in the maxillary process and the SOM (n = 3) (Fig. 1c,d). At E11.5, endogenous *Dlx5* expression was seen in the frontal bone primordium of the SOM (Fig. 1e). In the *NCC*^*Dlx5*^, *Dlx5* was expressed in the EMM besides the SOM (n = 3) at E11.5 (Fig. 1f, arrowhead). Therefore, *Dlx5* expression was successfully induced in NCCs, including EMM, in the *NCC*^*Dlx5*^.

We examined the NCC distribution in the *NCC*^*Dlx5*^ by whole-mount in situ hybridization (WISH) for *Snail family transcriptional repressor 1* (*Snai1*), a NCC specifier^1^ , at E9.0 (n = 4). *Snai1* expression was shown in a comparable pattern between the control and the *NCC*^*Dlx5*^ (Fig. 1g,h). We next examined the *Snai1* expression by section ISH (n = 3). We confirmed that post-migratory NCC-derived mesenchyme at the surrounding of the forebrain and the mandibular process similarly expressed *Snai1* in both of the control and the *NCC*^*Dlx5*^ (Fig. 1i,j). In the dorsal region of the rhombencephalon, *Snai1* expression was detected in migrating NCCs in the control and the *NCC*^*Dlx5*^ (Fig. 1k,l), the data revealed no difference between the two in this area. These results indicated that *Dlx5*-overexpression do not affect the migration and distribution of cranial NCCs.

### Predisposition of NCC differentiation in the ***NCC***^***Dlx5***^ mouse

**Figure 2:**
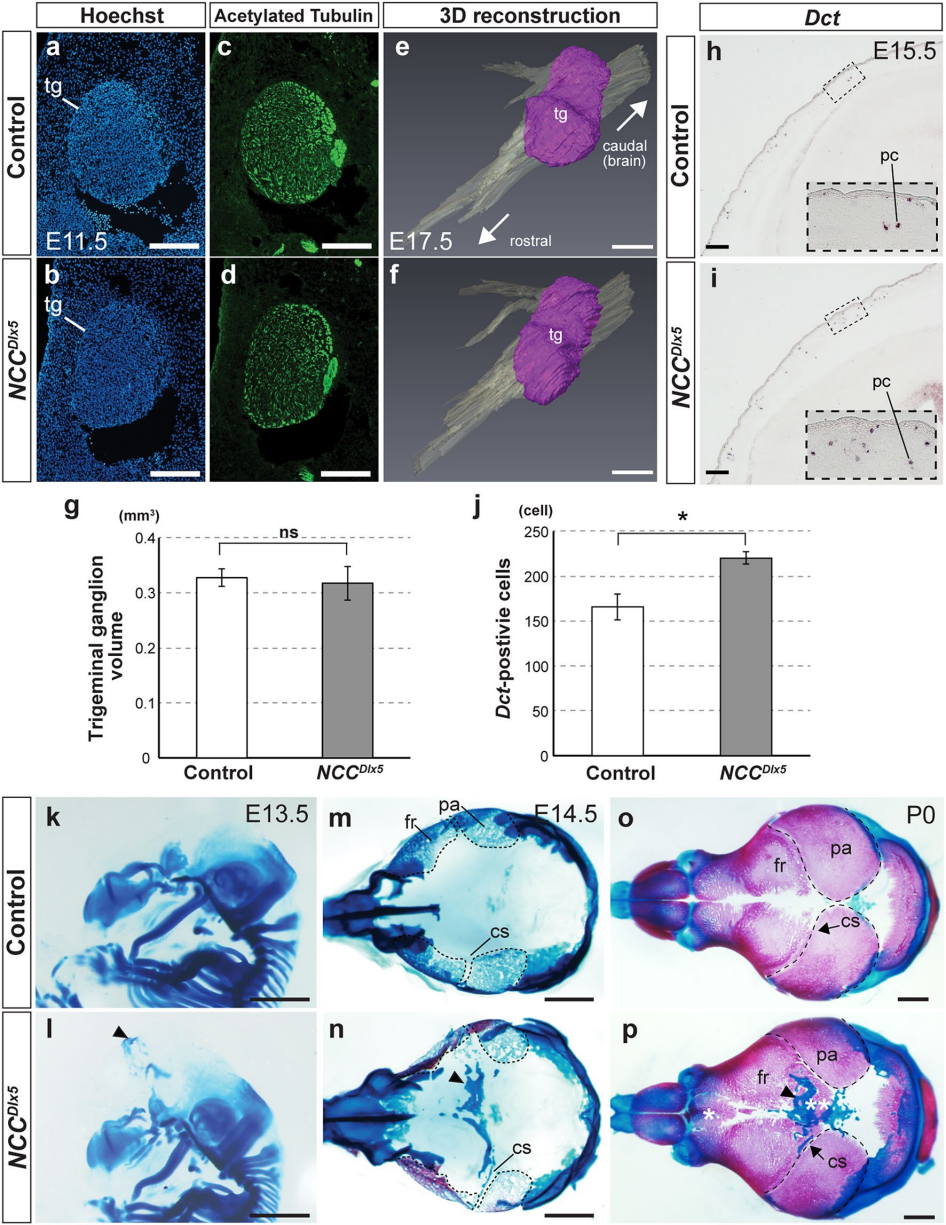
*Dlx5*-augmentation in NCCs modifies NCC-derivatives. (**a**–**d**) Immunohistochemical staining for acetylated tubulin (**c**, **d**), nuclear counterstained by Hoechst (**a**, **b**) in the trigeminal ganglion. (**e**, **f**) 3-D reconstruction of trigeminal ganglion of the control (**e**) and the *NCC*^*Dlx5*^ (**f**). (**g**) Statistical analysis of the trigeminal ganglion volume measured after reconstruction. (**h**, **i**) *Dct* localization, a pigment cell specifier, by ISH on frontal section of E15.5 heads. Insets in (**h**, **i**) are high magnified images of the boxed areas. (**j**) Statistical analysis of the number of *Dct*-positive cells. (**k**–**p**) Skeletal staining for cartilage (alcian blue) and bone (alizarin red). Lateral views at E13.5 (**k**, **l**), dorsal views (skull base removed) of calvaria at E14.5 (**m**, **n**) and P0 (**o**, **p**) of skeletal staining in the control and the *NCC*^*Dlx5*^. Ectopic cartilage is induced in the *NCC*^*Dlx5*^ (arrowheads in **l**, **n**, **p**) from E13.5. The developing heterotopic bone overlaps with the ectopic cartilage (double asterisks in **p**) at P0. Dashed lines contour cranial bones. Two-tailed t-test; *p < 0.05; ns, not significant. cs, coronal suture; fr, frontal bone; pa, parietal bone; pc, pigment cell; tg, trigeminal ganglion. Scale bar; 200 μm (**a**–**d**, **h**, **i**), 500 μm (**e**, **f**) and 1 mm (**k**–**p**).

*Dlx5* is normally expressed in the trigeminal ganglion^14^, and the size is reduced in *Dlx5* knock-out mice^7^. Acetylated tubulin staining at E11.5 demonstrated that neuron localization did not show obvious difference between the control and the *NCC*^*Dlx5*^ (n = 3) (Fig. 2a–d). Reconstructed trigeminal ganglion from the serial histological sections at E17.5 illustrated the similar shape and size of the control and the *NCC*^*Dlx5*^ (Fig. 2e,f) and no significant difference in volume (n = 3) (Fig. 2g). We also examined the pigment cell, another NCC-derivative^1^, by expression of *dopachrome tautomerase* (*Dct*)^34^ on frontal sections at E15.5 (n = 3) (Fig. 2h,i). The number of *Dct*-positive cells in the head dermis was significantly higher in the *NCC*^*Dlx5*^ than that of the control (*p* < 0.05) (Fig. 2j), suggesting that the NCC potential for pigment cell differentiation was enhanced by *Dlx5*-augmentation.

We next examined bone and cartilage formation in the calvarium. In the control, chondrocranium cartilages were observed at the skull base and lateral walls, while no cartilage and bone was observed at the apical part of the head at E13.5 and E14.5 (Fig. 2k,m). Interestingly, cartilage was formed at the vertex of the *NCC*^*Dlx5*^ (Fig. 2l,n). This calvarial cartilage was newly introduced to the region that usually has no cartilage, hence it is an ectopic cartilage^35^ (hereafter ec). The ec did not connect with cartilages at the skull base or the lateral wall and appeared like a bridge connecting bilateral hemispheres at E13.5 (n = 4) and E14.5 (n = 4) (Fig. 2l,n, arrowhead). As the frontal and parietal bones further developed at E14.5, the coronal suture was identified between the frontal bone and parietal bone in the control (Fig. 2m). The ec was seen anterior to the prospective coronal suture and seemed to outline the posterior border of NCC-derived frontal bone (Fig. 2n, arrowhead). The ec remained unossified (n = 5) (Fig. 2p, arrowhead), whereas no cartilage was detected at the same region in the control even at postnatal day 0 (P0) (Fig. 2o).

At P0, calvarial bone formation was distinct between the control and the *NCC*^*Dlx5*^. The frontal bone developed toward the midline, forming the interfrontal suture in the control (Fig. 2o). In the *NCC*^*Dlx5*^, not only ec but bony islands were also found in the interfrontal suture (Fig. 2p, asterisk) and the posterior part of frontal bone forming area (Fig. 2p, double asterisk). In some *NCC*^*Dlx5*^ mice, “patchy” bones with holes (Supplementary Fig. S1a,b) were formed randomly in the frontal bone and interfrontal area. These irregular bones seemed to fuse to the frontal bone, therefore we called them heterotopic bones^35^ (hereafter hb). In summary, *Dlx5* ectopic expression in head mesenchyme induced ec and hb formations. The development of the endogenous frontal bone was comparable between the control and the *NCC*^*Dlx5*^ at P0 (Fig. 2o,p). In the *NCC*^*Dlx5*^, *Dlx5* was not augmented in MES but the relative position of bilateral parietal bones in the cranium was abnormal at P0, and the anterior edge of parietal bone, which comprises the coronal suture was more inclined to the posterior (Fig. 2p). We reason that the inclination was caused by the appearance of ec and hb that interfered the normal parietal bone development.

Given the significant effects of *Dlx5* on NCC, we attempted to analyze MES-specific *Dlx5*-augmented mice by crossing *Mesp1-Cre* mice^36^ and *R26R*^*CAG-flox-Dlx5/*+^. Four litters at E11.5–13.5 were examined, but all *Mesp1-Cre;R26R*^*CAG-flox-Dlx5/*+^ fetuses were lethal, making following analyses impossible.

### NCC potential for chondrogenesis and osteogenesis was increased in the calvaria

Skeletal staining analysis of the *NCC*^*Dlx5*^ revealed that both chondrogenesis and osteogenesis were promoted simultaneously at the same region of the calvarium, which has not been reported in any other calvarial bone mutants. We thus further analysed the phenotype. Because the ec and hb were present at the NCC-MES junction, we first confirmed the cell origin of the misregulated structures. The NCC domain was visualized by *enhanced yellow fluorescent protein* (*EYFP*) in the *Wnt1-Cre;R26R*^*CAG-flox-Dlx5/EYFP*^ (*NCC*^*Dlx5/EYFP*^) and in the littermate control *Wnt1-Cre;R26R*^*EYFP/*+^ (*NCC*^*EYFP*^) at E17.5.

**Figure 3:**
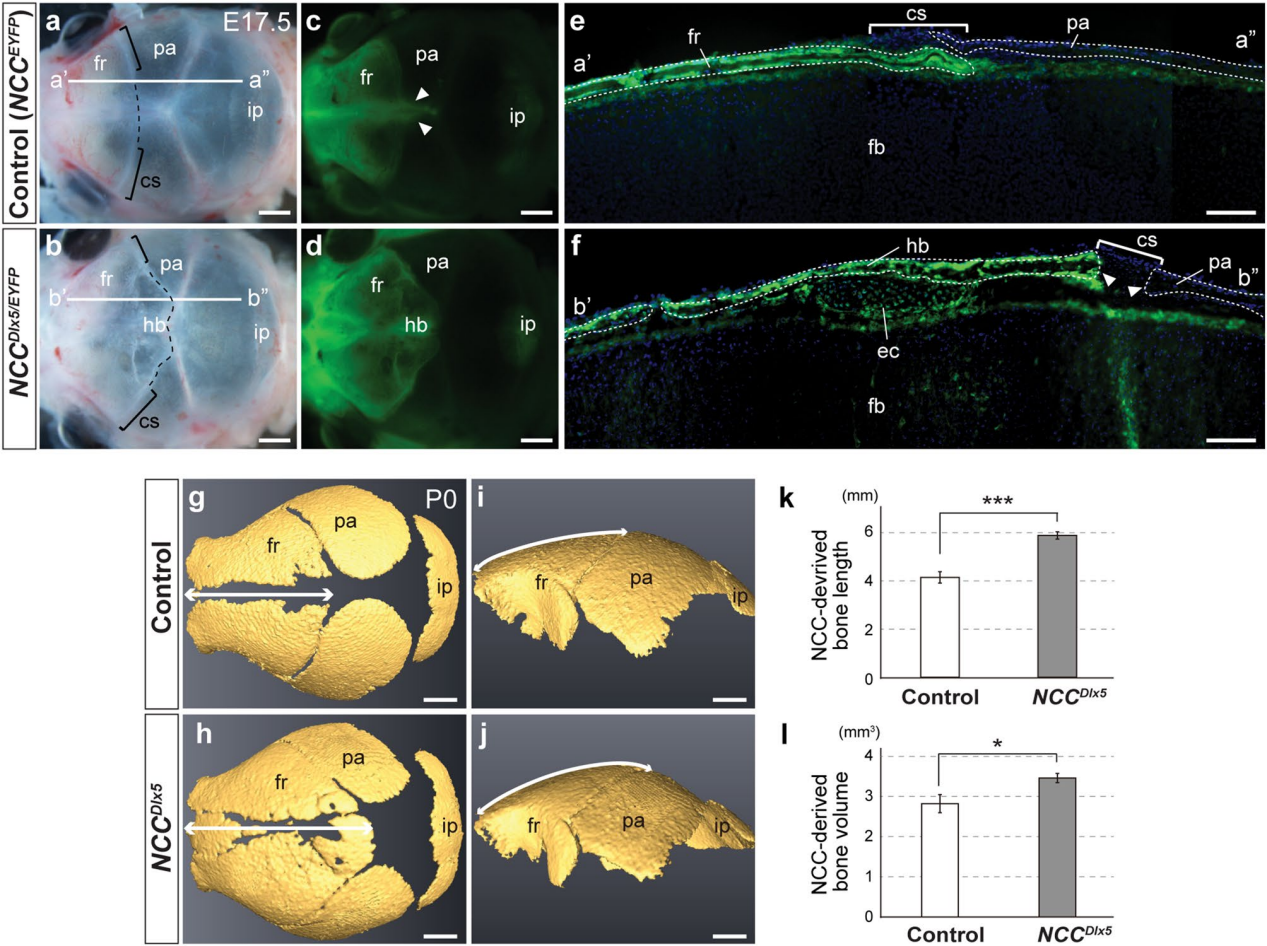
Ectopic cartilage and heterotopic bone in the *NCC*^*Dlx5*^ formed in NCC-derived head mesenchyme. Skull vault of E17.5 *NCC*^*EYFP*^ (**a**, **c**, **e**) and *NCC*^*Dlx5/EYFP*^ (**b**, **d, f**) with brightfield (**a**, **b**) and fluorescent (**c**–**f**) images. (**c**) and (**d**) are the fluorescent images of (**a**) and (**b**), respectively. Green fluorescence implies NCC originating cells. Brackets and dashed lines in dorsal views (**a, b**) indicate the coronal suture at the lateral side and the prospective coronal sutures at the apex, respectively. Arrowheads in (**c**) point to the “NCC tongue” between the parietal bones. (**e**, **f**) Parasagittal sections of the control (*NCC*^*EYFP*^) and mutant (*NCC*^*Dlx5/EYFP*^) at planes indicated in (**a**) (a'–a'') for (**e**) and (**b**) (b'–b'') for (**f**). Nuclei are counterstained by Hoechst. Brackets and arrowheads in (**e**, **f**) indicate the coronal sutures of the control and the opened end-to-end junction at the coronal suture of the mutant, respectively. (**g**–**j**) μCT images of calvarial bone of the control (**g**, **i**) and the *NCC*^*Dlx5*^ (**h**, **j**) at P0. Double-head arrows in (**g**–**j**) demonstrate the NCC-derived bone length to be measured. (**k, l**) Statistical analyses of NCC-derived bone length (**k**) and volume (**l**) (n = 3). Two-tailed t-test, *p < 0.05; ***p < 0.001. cs, coronal suture; ec, ectopic cartilage; fr, frontal bone; hb, heterotopic bone; ip, interparietal bone; pa, parietal bone. Scale bars; 1 mm (**a**–**d, g**–**j**) and 100 μm (**e**, **f**).

In bright-field images, the baso-lateral part of the coronal suture was comparable between the *NCC*^*EYFP*^ and the *NCC*^*Dlx5*/*EYFP*^ (n = 5) (Fig. 3a,b, brackets). However, the coronal suture at the vertex seemed to be shifted more posterior in the *NCC*^*Dlx5/EYFP*^ compared to the *NCC *^*EYFP*^ (Fig. 3a,b, dashed line). Under the fluorescent microscope, we found that the frontal bone of the *NCC*^*EYFP*^ was highlighted while the parietal bone was not, and the "NCC tongue"^3^ protruded to the sagittal suture (n = 5) (Fig. 3c, arrowheads). The ec and hb were formed within the fluorescent NCC-derived domain in the *NCC*^*Dlx5/EYFP*^ (n = 5) (Fig. 3d). In sagittal sections, the ec and hb were clearly detectable by fluorescence (n = 3) (Fig. 3f). The hb was formed in line with the frontal bone and parietal bone, and the ec was always seen underneath the bone-forming layer (Fig. 3f). In the *NCC*^*EYFP*^, osteogenic fronts of the frontal and parietal bones normally overlap at the coronal suture (Fig. 3e), however, in the *NCC*^*Dlx5/EYFP*^, the suture was established in a widely opened end-to-end type (Fig. 3f, arrowheads). Therefore, the extended NCC-derived area hosted the NCC-derived ec and hb, and the position of the coronal suture was shifted backwards. Computed X-ray microtomography (μCT) data of P0 (n = 3) revealed that the NCC-derived frontal bone length at the midline of calvaria in the *NCC*^*Dlx5*^ increased significantly by 18.5% compared to control (*p* < 0.001) (Fig. 3g–k). Furthermore, the NCC-derived calvarial bone volume rose by 10.2% (*p* < 0.05) (Fig. 3l). The volume of MES-derived parietal bone, meanwhile, did not significantly decrease (n = 3) (Supplementary Fig. S1c). Besides, the ec was found at the cranial vertex, the chondrogenic potential of the *NCC*^*Dlx5*^ obviously increased in this area. Thus, the NCC-derived apical head mesenchyme increased chondrogenic and osteogenic potentials in response to *Dlx5*-overexpression.

### The ec developed inside of the dura mater

**Figure 4:**
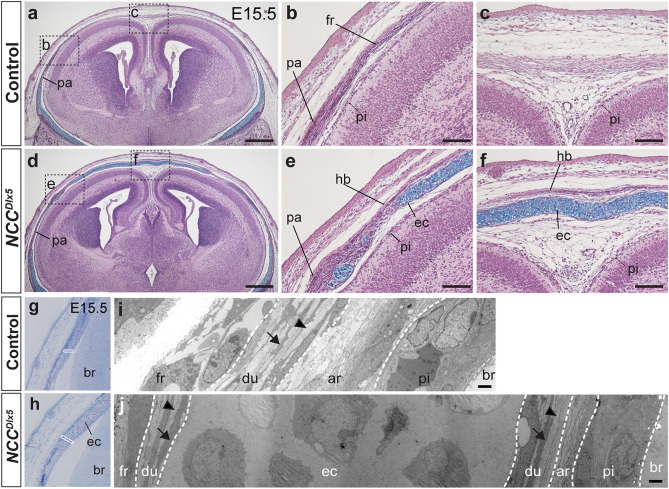
Histological analysis of the ectopic cartilage and heterotopic bone in the *NCC*^*Dlx5*^ at E15.5. (**a**–**f**) Frontal sections of E15.5 control (**a**–**c**) and *NCC*^*Dlx5*^ (**d**–**f**) stained by HE and Alcian blue. (**b**, **c**, **e**, **f**) are high magnification images of boxed areas in (**a**, **d**). Heterotopic bone is induced at the vertex of the *NCC*^*Dlx5*^, ectopic cartilage locates under the bone forming layer (**e**, **f**). (**g, h**) Semi-thin sections stained by toluidine blue on frontal sections of the control (**g**) and the *NCC*^*Dlx5*^ (**h**). (**i, j**) TEM analysis of the control (**i**) and the *NCC*^*Dlx5*^ (**j**) at the white boxed areas in (**g**, **h**). Arrows and arrowheads in (**i**, **j**) point to longitudinal-arranged fibroblasts and collagen fibrils, respectively. Ectopic cartilage appears in the meninges and is flanked by dura mater (**j**). ar, arachnoid mater; br, brain; du, dura mater; ec, ectopic cartilage; fr, frontal bone; hb, heterotopic bone; pa, parietal bone; pi, pia mater. Scale bars; 500 μm (**a**, **d**), 100 μm (**b**, **c**, **e**, **f**), 2 μm (**i**, **j**).

Histological analysis at E15.5 showed that cranial bones had not reached to the midline, and cartilage was absent in the vertex in the control (Fig. 4a–c). In the *NCC*^*Dlx5*^, the thickness of the ec was comparable to that of cranial base cartilages (n = 3) (Fig. 4d–f). The hb was ossified on top of the ec in the calvaria (Fig. 4d–f). As expected from the skeletal staining data (Fig. 2), the endogenous frontal bone and parietal bone of the *NCC*^*Dlx5*^ observed on HE sections illustrated similar bone quality in terms of thickness or degree of mineralization compared to the counterpart of the control (Supplementary Fig. S2). We conducted a more detailed investigation by using transmission electron microscope (TEM) at E15.5 (n = 3). Using toluidine blue stained semi-thin sections, we chose the relevant area of the control and the *NCC*^*Dlx5*^ for TEM analysis (Fig. 4g–j). In the control, the dura mater located just underneath the bone layer, characterized by longitudinally arranged fibroblast-like cells (Fig. 4i, arrow), and collagen bundles filling intercellular spaces (Fig. 4i, arrowhead)^37^. The arachnoid mater was clearly seen next to the dura mater, which contains more loosely attached cells, and numerous free ribosomes^28,37,38^ (Fig. 4i). In the *NCC*^*Dlx5*^, the ec occupied a large space between the bone and the brain (Fig. 4j). On its outer and inner surfaces, similar structures that had the characteristics of the dura mater were found (Fig. 4j). Besides, the arachnoid mater was recognized under the dura mater structure (Fig. 4j). Therefore, our histological analyses demonstrated that the ec developed within the dura mater.

### The ec and hb were derived from the EMM

It was reported that apical mesenchyme has both osteogenic and chondrogenic potential *in vitro*^39^. Double conditional knock-out of *Msh homeobox 1/2* (*Msx1/2*) in the mouse NCC (*Msx1/2*^*cko/cko*^) generated heterotopic bones from the EMM at abnormal positions including the suture area^32^. More recently, in vivo loss and gain of function experiments of *LIM homeobox transcription factor 1 beta* (*Lmx1b*), which is expressed in the EMM but not in the SOM, demonstrated the inhibitory function of *Lmx1b* on osteogenic specification in the EMM^27^. *Lmx1b* loss-of-function in head mesenchyme (*Lmx1b* LOF^HM^) induced osteogenic marker expression in the vertex mesenchyme, future interfrontal suture and expanded bone-forming area resulting in synostosis^27^. These previous studies suggest that the EMM has osteogenic potential, which is inhibited in normal context.

**Figure 5:**
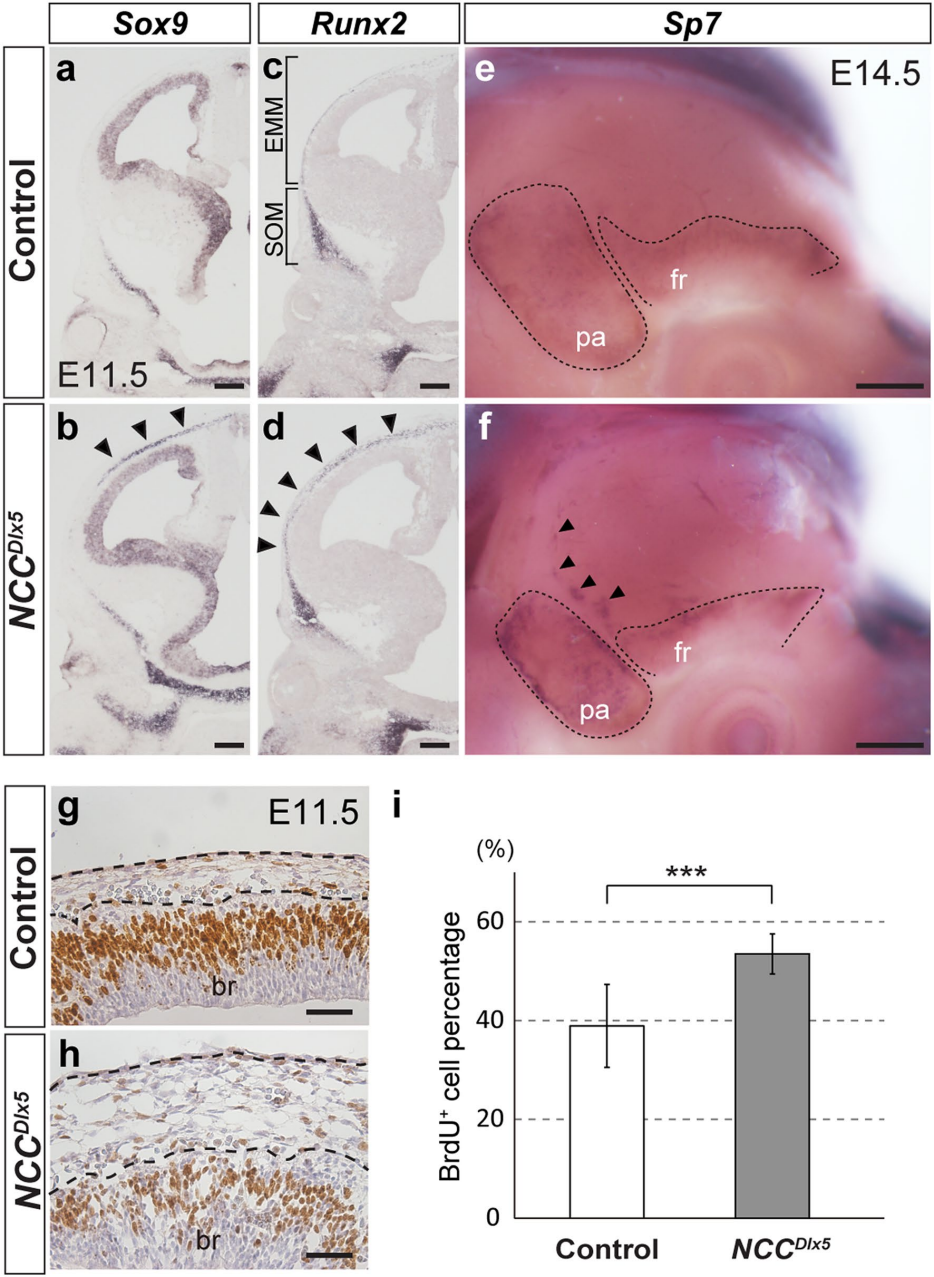
EMM developed the ectopic cartilage and heterotopic bone. (**a**–**d**) Expression of chondrocyte marker-*Sox9* (**a**, **b**) and osteoblast marker-*Runx2* (**c**, **d**) on frontal sections of E11.5 control (**a**, **c**) and *NCC*^*Dlx5*^ (**b**, **d**) by ISH. Arrowheads indicate ectopic expressions of *Sox9* (**b**) and *Runx2* (**d**) in the EMM of the *NCC*^*Dlx5*^. (**e, f**) Lateral views of *Sp7* expression, an early osteoblast marker and downstream of *Runx2*, by WISH on heads of E14.5 control (**e**) and *NCC*^*Dlx5*^ (**f**). Dotted lines in (**e**, **f**) outline developing frontal and parietal bones. Arrowheads in **f** point bony islands independent of the frontal bone primordium in the *NCC*^*Dlx5*^. (**g**, **h**) BrdU incorporation assay in the EMM at E11.5 of the control (**g**) and the *NCC*^*Dlx5*^ (**h**). BrdU positive cells were counted in the areas between the dashed lines of (**g**, **h)**. (**i**) Statistical analysis of the percentage of BrdU positive cells over the total number of EMM cells (n = 3). Two-tailed t-test,***p < 0.001. EMM, early migrating mesenchyme; fr, frontal bone; pa, parietal bone; SOM, supraorbital mesenchyme. Scale bars; 200 μm (**a**–**d**), 500 μm (**e**, **f**), 50 μm (**g**, **h**).

We explored the gene expression change that led to ec and hb formations. At E10.5, few mesenchymal cells were detected at the apical head, and histological difference between the control and the *NCC*^*Dlx5*^ was not noticed (Fig. 1a,b). We found that there were differences in gene expression as well as histology from E11.5. *SRY-Box transcription factor 9* (*Sox9*) and *Runt-related transcription factor 2* (*Runx2*) were used for evaluating mesenchymal condensation of cartilage and bone, respectively. At E11.5, *Sox9* and *Runx2* were substantially upregulated in the EMM region of the *NCC*^*Dlx5*^ compared to the control (Fig. 5a–d, arrowheads). Mesenchymal condensation for the ec and hb was found at E11.5, which was around the same time with the beginning of original cranial base and calvarial development. The ectopic *Sox9* expression domain was not connected to any part of the future skull base domain (n = 5) (Fig. 5b). This result confirmed that the ectopic *Sox9* expression was not due to the extension of skull base primordium. In contrast, *Runx2* expression in the EMM seemed to be continuous with the SOM by a thin expression line in the *NCC*^*Dlx5*^ (n = 4) (Fig. 5c,d). To test whether the developing hb was independent of the SOM, we examined expression of *Sp7*, an early osteoblast marker and downstream of *Runx2*, at E14.5 by WISH (n = 5). The development of frontal and parietal bones was visualized by *Sp7* expressing domain at this stage (Fig. 5e,f, dotted line). In the EMM area of the *NCC*^*Dlx5*^, several *Sp7* expression islands were independent of the SOM (Fig. 5f, arrowheads). These results strongly suggested that the hb in the *NCC*^*Dlx5*^ is formed in the EMM and independent of the endogenous frontal bone.

The EMM layer was thickened in the *NCC*^*Dlx5*^ at E11.5, which contained expanded *Sox9* and *Runx2* expression domains (Fig. 5g,h). Our BrdU incorporation assay showed a significantly increased BrdU^+^ cells in the EMM of E11.5 *NCC*^*Dlx5*^ (n = 3, *p* < 0.001) (Fig. 5i). Immunohistochemical (IHC) staining for cell death showed no signals in the EMM of both the control and the *NCC*^*Dlx5*^ at E11.5 (n = 3) (Supplementary Fig. S3). Therefore, the thickened EMM in the *NCC*^*Dlx5*^ was caused by increased cell proliferation.

### Early development of the EMM in the control and the ***NCC***^***Dlx5***^

**Figure 6:**
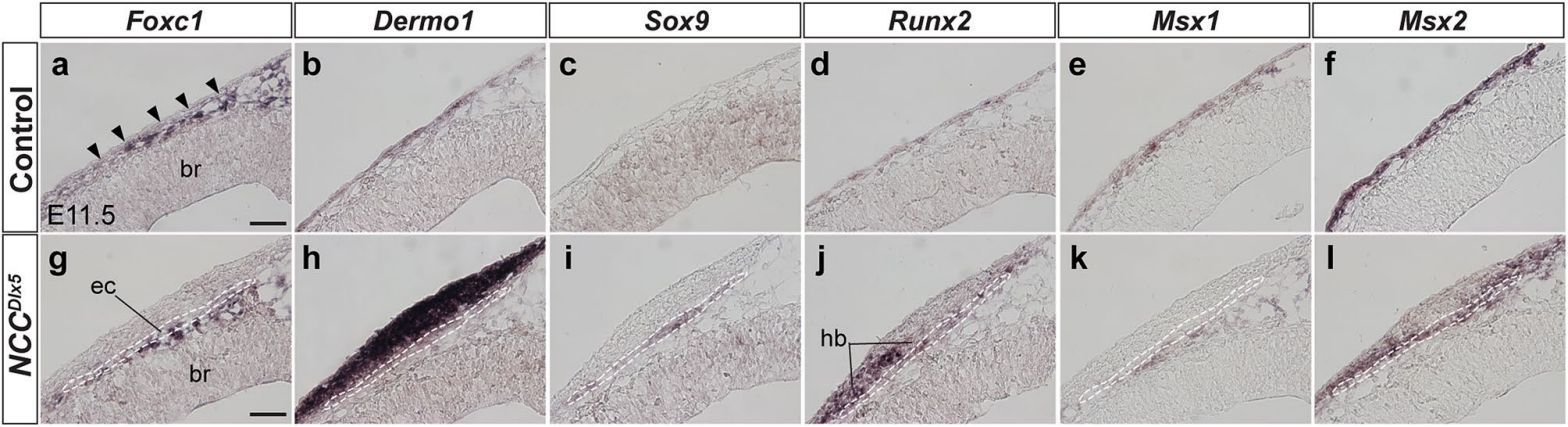
Gene expression patterns in the EMM at E11.5. Expression pattern of *Foxc1-*meningeal marker (**a**, **g**), *Dermo1*-dermal marker (**b**, **h**), *Sox9*-chondrocyte marker (**c**, **i**), *Runx2*-osteoblast marker (**d**, **j**), and osteogenic regulators: *Msx1* (**e**, **k**), *Msx2* (**f**, **l**) on frontal sections of E11.5 control (**a**–**f**) and *NCC*^*Dlx5*^ **(g–l)** by ISH. Arrowheads in (**a**) point cells under the epidermis that weakly express *Foxc1*. *Foxc1* and *Dermo1* demonstrate complementary expression in head mesenchyme in the control (**a**, **b**), *Dermo1* and *Runx2* are co-expressed in a thin cell layer under the epidermis (**b**, **d**). *Msx1* is expressed in the whole head mesenchyme (**e**), while *Msx2* is mainly expressed at the outer layer of head mesenchyme (**f**). In the *NCC*^*Dlx5*^, *Sox9* expression in EMM illustrates the forming ectopic cartilage (**i**). *Foxc1* is expressed in the meninges and faintly in the ectopic cartilage (**g**). *Dermo1* and *Runx2* are upregulated; *Runx2* expression is enclosed by *Dermo1* expression (**h**, **j**). *Msx1* is not expressed in ectopic cartilage and heterotopic bone, whereas *Msx2* is expressed in both misregulated structures. Dashed lines in (**g**–**l**) outline the ectopic cartilage forming region estimated by *Sox9* expression (**i**). br, brain; ec, ectopic cartilage; hb, heterotopic bone. Scale bars; 50 μm.

We examined gene expression in early development of the ec and hb (n = 4) at E11.5. In the control, expression of *Forkhead Box C1* (*Foxc1*), transcribed in all three meningeal layers^40^, was broadly detected in the mesenchyme, but the signal was not seen or at much lower levels just underneath the epidermis at E11.5 (Fig. 6a, arrowheads). Importantly, expression domains of *Foxc1* and *Dermo1*, molecular markers for the meninges and the dermis^41,42^, respectively, were mutually exclusive (Fig. 6a,b). Control mice showed no expression of *Sox9* in the EMM (Fig. 6c), but *Runx2* was expressed in the *Dermo1* expressing mesenchyme as a thin layer (Fig. 6b,d). *Msx1* expression was found in the whole head mesenchyme (Fig. 6e). *Msx2* expression domain was localized to the outer layer of the EMM, including a part of the meninges and the dermis (Fig. 6f, compared to 6a,b).

In the *NCC*^*Dlx5*^, the *Foxc1* expression domain appeared to contain the ec primordium marked by *Sox9* expression (Fig. 6g,i), which is consistent with the phenotype in which the ec is surrounded by the dura mater (Fig. 4j). Remarkably, *Dermo1* expression was highly upregulated in the *NCC*^*Dlx5*^ compared to the control (Fig. 6h), indicating that *Dlx5*-augmentation enhanced the dermis formation. *Runx2* expression of the *NCC*^*Dlx5*^ was more evident compared to the control (Fig. 6d,j). *Sox9* expression domain was included in the *Runx2* domain (Fig. 6i,j). Because the hb developed outside of the ec (Fig. 4f), *Runx2* expressing cells outside of *Sox9*-positive layer were thought to differentiate into osteoblasts. Importantly, these osteoblasts also expressed *Dermo1* (Fig. 6h,j), suggesting that the hb was derived from the dermal layer. In contrast, the ec shown by *Sox9* expressing domain seemed not to show *Dermo1* expression (Fig. 6h,i).

Moreover, *Msx1* expression was present in the arachnoid and the pia mater, and was not expressed in other parts of the EMM (Fig. 6k). *Msx2* was downregulated at some areas of head mesenchyme, however, expressed in the ec and hb (Fig. 6l). Since *Lmx1b* loss-of-function induced hb formation^27^, we also examined *Lmx1b* expression. *Lmx1b* was expressed in both the ec and hb (Supplementary Fig. S4), suggesting that since *Dlx5* is a downstream of *Lmx1b*^27^, *Dlx5*-overexpression does not affect *Lmx1b* expression.

### PDGFRα, WNT/β-catenin and ***Bmp2*** signals are upregulated in the*** NCC***^***Dlx5***^

**Figure 7:**
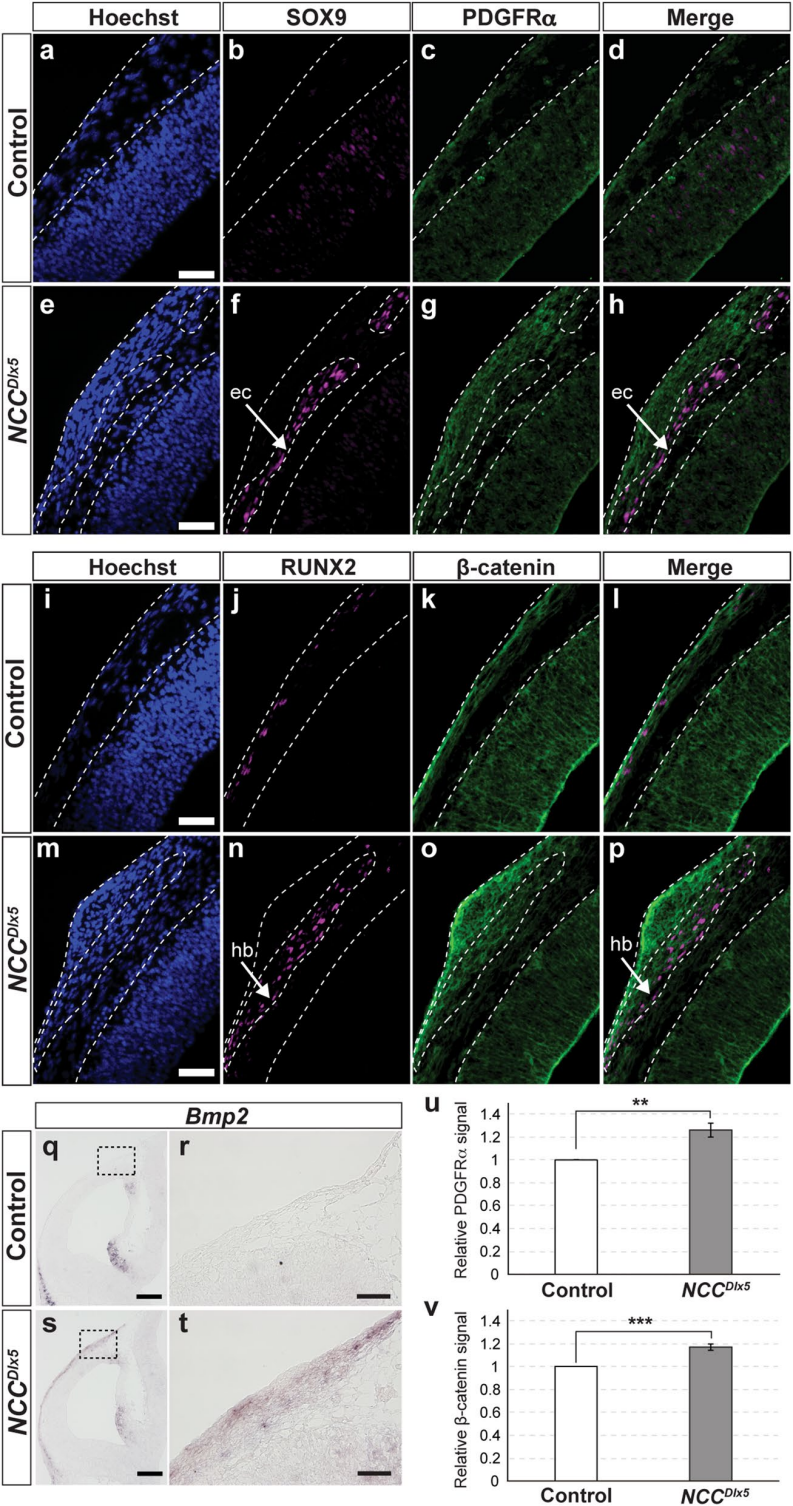
PDGFRα, β-catenin and *Bmp2* signals in the EMM at E11.5. (**a**–**h**) Double immunofluorescent staining for SOX9 and PDGFRα on frontal sections at E11.5, nuclear counterstained by Hoechst of the control (**a**–**d**) and the *NCC*^*Dlx5*^ (**e**–**h**). Arrows in **f**, **h** point to the ectopic cartilage. PDGFRα signal is shown in the SOX9-positive area (**h**). (**i**–**p**) Double immunofluorescent staining for RUNX2 and β-catenin on frontal sections at E11.5, nuclear counterstained by Hoechst of the control (**i**–**l**) and the *NCC*^*Dlx5*^ (**m**–**p**). Arrows in (**n**, **p**) point to the heterotopic bone. β-catenin signal is shown in the RUNX2-positive area (**p**). (**q**–**t**) *Bmp2* expression by ISH on frontal sections at E11.5 of the control (**q**, **r**) and the *NCC*^*Dlx5*^ (**s**, **t**). (**r**, **t**) are high magnified images of the boxed area in (**q**, **s**). (**u**, **v**) Semi-quantification analysis on immunofluorescent staining for PDGFRα and β-catenin. Two-tailed t-test;**p < 0.01;***p < 0.001. ec, ectopic cartilage; hb, heterotopic bone. Scale bars; 50 μm (**a**, **e**, **i**, **m**, **r**, **t**); 200 μm (**q**, **s**).

*Platelet-derived growth factor receptor Alpha (Pdgfra)* augmented in NCCs generated ec at the coronal suture, which was similar to the ec of the *NCC*^*Dlx5*^^43^. We performed double imunnofluorescent staining for PDGFRα and SOX9 at E11.5 (n = 3), PDGFRα was present in the outer portion of the EMM in the control, while SOX9 signal was not detected (Fig. 7a–d). In the *NCC*^*Dlx5*^, PDGFRα expression levels were more intensive in the dermal and SOX9-positive layers (Fig. 7e–h). Semi-quantitative analysis on immunofluorescent staining showed that PDGFRα signal was intensified by *Dlx5*-augmentation (Fig. 7u). WNT/β-catenin signalling induces osteoblast differentiation in intramembranous ossification^44,45^. The conditional β-catenin loss-of-function in the dermis using *Dermo1-Cre* or *Engrailed1-Cre* driver resulted in the loss of dermis and cranial bones. Instead, cartilages were induced between the epidermis and the thinner meninges^42^. Reversely, the *NCC*^*Dlx5*^ had thickened dermis (Fig. 6h) and hb (Fig. 4f). We conducted double immunofluorescent staining for β-catenin and RUNX2 signals at E11.5 (n = 3). In the control, β-catenin and RUNX2 were sparsely expressed below the epidermis (Fig. 7i–l). In the *NCC*^*Dlx5*^, RUNX2 signal illustrated the hb (Fig. 7m,n), β-catenin signal in the dermis and hb forming area were clearly upregulated (Fig. 7m–p). Semi-quantitative analysis for β-catenin showed that the signal was significantly upregulated by *Dlx5*-augmentation (Fig. 7v).

*Bone morphogenetic protein (Bmp)* is involved in hb formation in the interfrontal suture^32,46^ and it regulates *Runx2* expression through DLX5^47^. Our data showed that *Bmp2* was not expressed in the control head mesenchyme at E11.5 (Fig. 7q,r). However, it was ectopically induced in hb forming area in the *NCC*^*Dlx5*^ (n = 3) (Fig. 7s,t).

## Discussion

*Dlx5* expression in the NCC is involved in jaw patterning. Aside from that, *Dlx5* is expressed in the several NCC-derived head components and is related to their differentiation^1^, thus, we further examined predisposition of the NCC affected by *Dlx5*-augmentation. Investigations of *Snai1* expression indicated that the migration and distribution of NCCs were unaffected by *Dlx5*-overexpression (Fig. 1). There was little effect on trigeminal ganglion development, but the number of pigment cells was increased in the *NCC*^*Dlx5*^ (Fig. 2), which possibly corresponds to enhanced dermal cell proliferation in the *NCC*^*Dlx5*^ (Figs. 5h, 6h). Despite the extra skeletogenesis in the *NCC*^*Dlx5*^, non-skeletal NCC-derivatives such as trigeminal ganglion, dermis, and pigment cells were not attenuated, suggesting that NCCs did not fluctuate between non-skeletogenic and skeletogenic fates.

Previous reports showed that modifications in molecular cascades in mouse head mesenchyme resulted in either ec or hb formation in the skull vault. In particular, *Msx1/2*^*cko/cko*^ and *Lmx1b* LOF^HM^ caused hb formation at the posterior of the frontal bone similar to the *NCC*^*Dlx5*^, but ec formation was not reported in those mutants^27,32^. By contrast, *Pdgfra* upregulation in NCCs generated ec in the coronal suture, meanwhile, the frontal bone appeared unchanged^43^. Some mutants demonstrated that cartilages replaced calvarial bones in mice, such as β-catenin knock-out^42,45^ and *fibroblast growth factor 8 (Fgf8)* gain-of-function^48^. Therefore, chondrogenesis could be upregulated at the expense of osteogenesis. In this study, we showed that chondrogenesis and osteogenesis were promoted simultaneously in the *NCC*^*Dlx5*^ calvaria (Fig. 2). Ec and hb formation in the *NCC*^*Dlx5*^ occurred in the meningeal and dermal layers of the EMM, respectively (Figs. 4, 6). We also found that, in normal development, the EMM seems to be committed to dermal and meningeal layers by E11.5 (Fig. 6). The distinct cell differentiation of apical head mesenchyme in response to *Dlx5*-augmentation in NCCs strongly support the idea that there are different cell populations in the EMM by this stage.

The development of the ec in dura mater layer highly suggests that head mesenchyme which was originally destined to be meningeal precursor cells could be turned into chondrocytes. The ec formation within the dura mater was previously reported; residual cartilages are occasionally formed above the trigeminal ganglia in mammals^49^, and the pila antotica near the ala temporalis in therians develops inside the dura mater as atavistic relics^50^. In the clinical aspect, some meningeal chondrosarcomas, which are tumours containing cartilaginous islands, in the dura mater are reported^51^. Additionally, when the dura mater is transplanted to the trunk in contact with mesodermal elements, the transplant sometimes develops cartilage^52^. In addition, explants of mouse mesenchymal cells from the vertex area of the head (corresponding to the EMM) at E12.5–14.0 show the potential for bone and cartilage formations^39^. These reports support our idea of dura-to-cartilage transformation.

**Figure 8:**
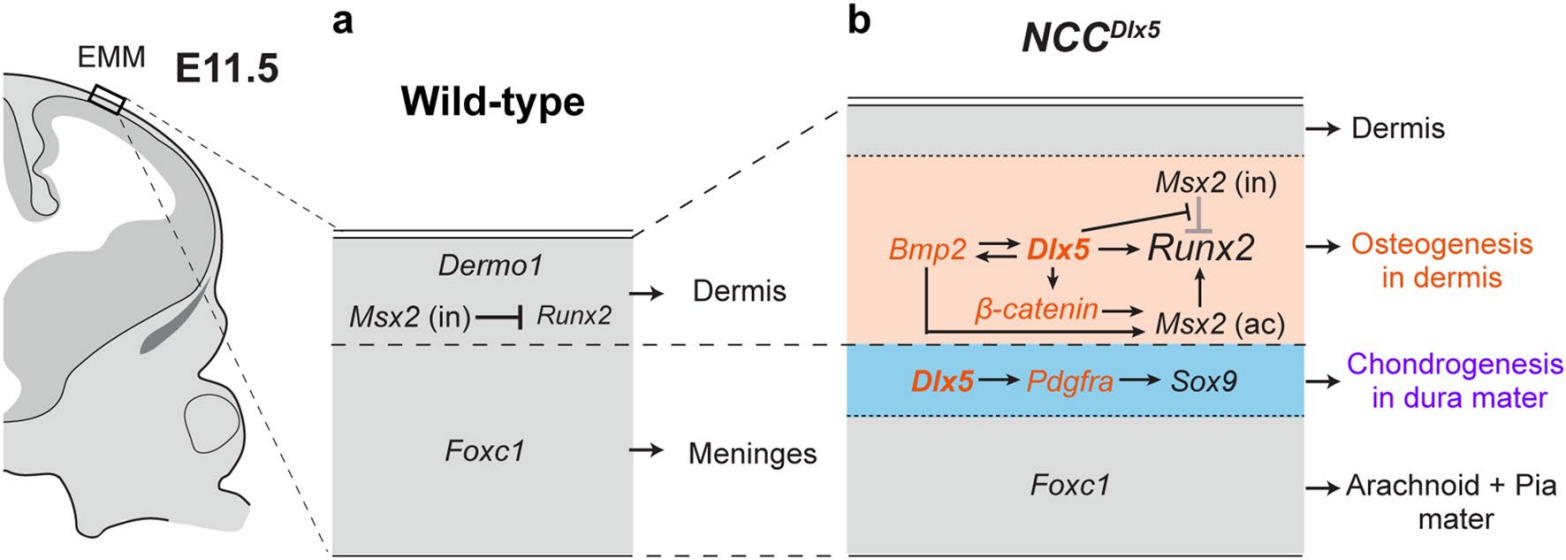
Proposed regulatory molecular cascades in EMM development at E11.5. In the control (**a**), the EMM develops into dermal and meningeal layers. The dermis is marked by a dermal marker-*Dermo1*. *Runx2* and *Msx2* are expressed in the dermis and *Msx2* [*Msx2* (in)] inhibits osteogenic induction of *Runx2*, consequently bone is not formed in the dermis. *Foxc1* is expressed underneath the dermal layer to induce meningeal cell differentiation. In the *NCC*^*Dlx5*^ (**b**), *Dlx5* upregulates *Pdgfra* in the dura mater to generate the ectopic cartilage at the interfrontal and NCC-MES boundary area. In the dermis layer, *Dlx5* inhibits the anti-osteogenic function of *Msx2* [*Msx2* (in)] but support the pro-osteogenic function of *Msx2* [*Msx2* (ac)] by upregulating *Bmp2* and β-catenin, resulting in *Runx2* upregulation. Consequently, osteogenesis is activated in the apical head mesenchyme to form the heterotopic bone. ac, activator; in, inhibitor.

In the *NCC*^*Dlx5*^, the ec develops in the frontal bone area in proximity to the coronal suture, which is the NCC-MES boundary, and some small cartilages in the interfrontal suture. Loss of β-catenin is one of the causes of ec induction during calvarial development^42^. However, β-catenin was detected in the ec forming area in the *NCC*^*Dlx5*^ (Fig. 7f,o), suggesting that formation of ec was not attributed to change in WNT/β-catenin signalling. *Pdgfra* upregulation in the NCC in mice exhibits ec formation in the coronal and interfrontal suture, which is similar to the ec in the *NCC*^*Dlx5*^^43^. We found that PDGFRα signal was increased in the *NCC*^*Dlx5*^. Although the layer of ec formation was not studied in the *Pdgfra* mutant^43^, it is suggested that the interfrontal area, the NCC-MES boundary show potential for cartilage differentiation when stimulated by PDGFRα (Fig. 8b).

Our gene expression analyses suggest that, in the control, *Runx2*-expressing layer just below the epidermis in the EMM does not associate with the meninges at E11.5. The *Runx2*-expressing layer is present within the expression domain of *Dermo1,* a dermal cell marker, and this expression domain does not express *Foxc1*, a meningeal marker. Based on these results, we propose that the cells expressing both *Runx2* and *Dermo1* in the EMM are dermal progenitors (Fig. 8a). Furthermore, the *Runx2* expression in mesenchymal cells suggests the intrinsic osteogenic potential of the dermis. Anti-osteogenic functions of *Msx2* and *Lmx1b* are likely to suppress the osteogenic potential in the EMM^27,32^.

*Msx2* can act as either osteogenic inhibitor or activator (Fig. 8, *Msx2* (in), *Msx2* (ac))^53^. MSX2 inhibits *Runx2* transcriptional activity^54,55^, and competes with RUNX2 in binding to a regulatory sequence of *Osteocalcin* (*Ocn*), an osteogenic induction gene^56^ (*Msx2* (in)). The anti-osteogenic activity of *Msx1* and *Msx2* in the EMM explained the hb in the *Msx1/2*^*cko/cko*^ at the early stage of calvarial development (~ E12.5)^32^. In the *Lmx1b* LOF^HM^, *Msx2* expression was downregulated in the EMM, and the phenotype could be explained similarly to the *Msx1/2*^*cko/cko*^^27^. We thus propose that *Msx2* inhibits *Runx2* osteogenic induction in the dermis in normal situation (Fig. 8a), and possibly *Msx1* is also related to this function to some extent.

Contrary to the anti-osteogenic function above mentioned, *Msx2* has been shown to promote both proliferation (undifferentiated condition) and differentiation of osteoblast lineage cells^57,58^ (*Msx2* (ac)). Despite the similar hb formation to the *Msx1/2*^*cko/cko*^, *Msx2* was expressed in the hb forming region in the *NCC*^*Dlx5*^ (Fig. 6). *Dlx5* is an important factor to antagonize *Msx2* anti-osteogenic function^55,59,60^, it therefore seems that *Dlx5*-overexpression suppressed the anti-osteogenic function of *Msx2* in the *NCC*^*Dlx5*^ (Fig. 8b). By contrast, *Dlx5* appears to promote the pro-osteogenic function of *Msx2* in concert with other osteoblast activators such as *Bmp2* and β-catenin (Fig. 8b, *Msx2*(ac)). *Dlx5* is a downstream target of *Bmp2*^47^, and we observed increased *Bmp2* expression in head mesenchyme of the *NCC*^*Dlx5*^. These results suggest that *Dlx5* activates *Bmp2* through positive feedback. Since *Msx2* is also a main downstream of *Bmp2*^61^, maintenance of *Msx2* expression in the *NCC*^*Dlx5*^ could be caused by *Bmp2* induction. WNT/β-catenin promotes intramembranous bone formation and dermal layer differentiation^42^. We demonstrated that β-catenin levels were increased in the forming hb and probably associated with the enhanced dermis differentiation (Fig. 7m–p). Our data are consistent with previous reports that *Bmp2* and β-catenin synergistically induce *Msx2*^62,63^. *Bmp2* upregulation in the *NCC*^*Dlx5*^ is consistent with *Msx1/Msx2*^*cko/cko*^ and *Lmx1b* LOF^HM^ mutants^27,32^. Altogether, the hb formation in the *NCC*^*Dlx5*^ was caused by enhanced osteogenic induction of *Bmp2* and β-catenin signalling pathways that involve *Msx2* (Fig. 8b). It will be intriguing to clarify in more detail about the molecular mechanism for the phenotype, involved in the dual function of *Msx2* in the future.

It was reported that *Lmx1b* prevents ossification of EMM from E9.5^27^. In this study, we found that expression patterns of *Dermo1* and *Foxc1* are mutually complementary at E11.5 in the control (Fig. 6a,b). Taken together, we propose that after non-skeletogenic commitment, the EMM is divided into two populations, dermal and meningeal layers by E11.5 in normal development (Fig. 8a). However, this commitment is not irreversible because the cell fate can be altered to cartilage and bone by responding to pro-skeletogenic signals such as augmentation of *Dlx5*. Given that the EMM has differentiation potentials to both cartilage and bone, it should be carefully evaluated which mesenchyme (EMM or SOM) contributes to ectopic and heterotopic skeletogenesis in the calvarium when mutant mice are examined.

Apical-basal patterning in cranial development by the interaction between the EMM and SOM has yet to be fully elucidated. Although the molecular basis of EMM differentiation in its early development is still unknown, our findings provide a more detailed picture of the EMM sublayers together with their potentials, shedding light on developmental mechanisms of cranial development.

## Materials and methods

### Mice

Mice with *Dlx5* conditional expression reporter allele by utilizing Cre-LoxP recombination system, *R26R*^*CAG-flox-Dlx5/*+^, were described previously^11^. *R26R*^*CAG-flox-Dlx5/*+^ mice were maintained on ICR genetic background. *Wnt1-Cre* (#022501) mice were obtained from the Jackson Laboratory (#022501, Maine, USA) and maintained on ICR genetic background. *Wnt1-Cre* driver targets the NCC^64^. *R26R*^*CAG-flox-Dlx5/*+^ mice were crossed with *Wnt1-Cre* mice to constitutively activate *Dlx5* expression in NCC. *Wnt1-Cre;R26R*^*CAG-flox-Dlx5/*+^ mice were used as the mutant, *NCC*^*Dlx5*^, and mice without *Wnt1-Cre* or *R26R*^*CAG-flox-Dlx5/*+^ allele were studied as the control. *R26R*^*lacZ*^ mice were described previously^65^. β-galactosidase staining of *Wnt-Cre;R26R*^*lacZ/*+^ (*NCC*^*LacZ*^) visualizes the NCC. *Wnt1-Cre;R26R*^*EYFP/*+^ mice expressing *enhanced yellow fluorescent protein (EYFP)* were crossed with *R26R*^*CAG-flox-Dlx5/*+^ mice to generate *Wnt1-Cre;R26R*^*CAG-flox-Dlx5/EYFP*^ (*NCC*^*Dlx5/EYFP*^), in which NCC expresses *EYFP* with *NCC*^*Dlx5*^ phenotype. The *Wnt1-Cre;R26R*^*EYFP/*+^ (*NCC*^*EYFP*^) control littermates express *EYFP* in the NCC. The morning on which a vaginal plug was found was designated as embryonic day 0 (E0). Animal procedures were approved by the Institutional Animal Care and Use Committee of Tokyo Medical and Dental University (0170238A, A2018-48C, A2019-060A) and the University of Tokyo (P19-043). All experiments were carried out in accordance with the relevant guidelines and regulations of the Tokyo Medical and Dental University and the University of Tokyo. Animal studies were conducted following the ARRIVE guidelines.

### In situ hybridization (ISH)

Embryos of E9.0–12.5 were collected in ice-cold phosphate-buffered saline (PBS). Samples were fixed with 4% paraformaldehyde/PBS overnight at 4 °C. For frozen sections, samples were incubated in 25% sucrose in PBS, finally embedding in O.C.T. compound (Sakura Finetek, Japan) and stored at − 80 °C. Heads of E15.5 were frozen freshly in O.C.T. compound. Frozen sections were cut at 12 μm thickness (Leica CM1850, Germany). For whole-mount in situ hybridization (WISH) purposes, the fixed samples were dehydrated in a graded series of methanol and stored in 100% methanol at − 20 °C. DNA fragments of mouse *Bmp2*, *Dct*, *Dlx5*, *Dermo1*, *Foxc1*, *Lmx1b*, *Msx1*, *Msx2*, *Runx2*, *Snai1*, *Sox9*, and *Sp7* shown in Supplementary Table S1 were subcloned into the pGEM-easy vector (Promega, USA), or pCRII vector (Invitrogen, USA). Digoxigenin (DIG)-labelled RNA probes complementary to the cDNA were synthesized by Sp6 or T7 RNA polymerase (Roche Diagnostics, Germany) and used for section or whole-mount in situ hybridization. Gene expression was visualized by nitro blue tetrazolium chloride and 5-Bromo-4-chloro-3-indolyl phosphate (Roche Diagnostics, Germany). In situ hybridization experiments were performed at least three times on different samples.

### Immunohistochemical (IHC) and immunofluorescent staining

Bromodeoxyuridine (BrdU) (Roche Diagnostics) was used for BrdU cell proliferating assay. BrdU in PBS was injected intraperitoneally to the pregnant mice at a dose of 100 mg BrdU/kg body weight one hour before dissection. E11.5 fetal heads were collected, fixed with 4% PFA, and prepared to be embedded in the O.C.T. compound. Other immunohistochemical analyses were performed on untreated fixed samples. Samples were cut at 12 μm. Sections were incubated with mouse anti-BrdU (1:200, 11170376001, Roche Diagnostics), rabbit anti-cleaved caspase3 (1:200, #9661, Cell Signaling Technology), or anti-Acetylated Tubulin (1:200, T7451, Sigma-Aldrich), double immunohistochemical staining with mixtures of rat anti-PDGFRα (1:500, 14-1401, eBioscience) and rabbit anti-SOX9 (1:1500, cat.#AB5535, Millipore), mouse anti- β-catenin (1:500, 610153, BD BioSciences) and rabbit anti-RUNX2 (1:150, #12556, Cell Signaling Technology). SOX9 and RUNX2 fluorescence signals were detected by anti-Rabbit Alexa Fluor 555 (1:300, A31572, Molecular Probes). Other stainings were processed with biotinylated anti-mouse IgG (1:200, ZA0409, Vector Laboratories), biotinylated anti-rabbit IgG (1:200, ZB1007, Vector Laboratories), biotinylated anti-rat IgG (1:200, BA-4001, Vector Laboratories) followed by Avidin–biotin complex (Vectastain) and 3,3′-diaminobenzidine (Sigma-Aldrich), or Alexa Fluor 488 streptavidin conjugate (1:300, S11223, Molecular Probes). IHC experiments were conducted at least three times on different samples.

### Semi-quantification of ISH, IHC and immunofluorescent staining

The number of *Dct*-positive cells was the total number counted in the EMM of three sections: middle of the eye, back of the eye, and behind the eye, at 10× magnification (n = 3). The percentage of BrdU positive cells was calculated by the number of BrdU positive cells divided by the total number of cells in the designated area, in four representative views at 40× magnification of each *NCC*^*Dlx5*^ mouse (n = 3) and four corresponding views of each control (n = 3). Fluorescent staining experiments were conducted with negative control sections without the primary antibody. Fluorescent images were treated equally with reference to the negative control sections by Photoshop (Adobe, USA). Semi-quantification of fluorescent signals was processed by ImageJ, relative signal was compared by a ratio of mean brightness values (brightness per area) of the *NCC*^*Dlx5*^ to the control (n = 3).

### 3-Dimension reconstruction of the trigeminal ganglion

Heads of E17.5 fetuses of the *Wnt1-Cre;R26R*^*CAG-flox-Dlx5/*+^ and the control were embedded in O.C.T. compound, sectioned at 12 μm thickness, and stained by Mayer’s Hematoxylin and 1% Eosin Y solution (HE) (Muto Pure Chemical, Japan). 3-Dimension structures were constructed from serial histological sections by Avizo 6.3 (Visualization Sciences Group, USA). Volume of the trigeminal ganglion of the control (n = 3) and the *NCC*^*Dlx5*^ (n = 3) was measured after reconstruction.

### Alizarin red and alcian blue skeletal staining

Post-natal day 0 (P0) mice were skinned, followed by fixation in 96% ethanol for one week. Skeletal staining was performed in a mixture of 0.02% alcian blue (Sigma, 05500-10G), 0.005% alizarin red (Wako, 013–25452), 5% acetic acid in 70% ethanol for three days with rocking at room temperature. Samples were then washed by distilled water and optically cleared by glycerol in 0.5% KOH until the bone and cartilage were visible.

### Computed X-ray microtomography (μCT)

Heads of P0 mice were fixed in 70% ethanol overnight. μCT was taken by inspeXio SMX100CT (Shimadzu, Japan). The data were analyzed by Avizo 6.3. μCT scans were uploaded to Avizo 6.3 as DICOM files and visualized using Isosurface in Avizo 6.3. The NCC-derived bone length was measured in three-dimension at the midline, tracing the top of the calvaria (Fig. 3g–j, n = 3). The bone volume was calculated by Avizo 6.3, including all the bone components within the frontal bone forming area.

### Histological analysis

Heads of fetuses at E15.5 were fixed in Bouin’s fixative solution for 48 h. The samples were washed by 70% ethanol, then dehydrated in a gradient of ethanol until 100%, followed by xylene treatment and embedded in paraffin. Sections were cut at 5 μm thickness (Leica RM2235, Germany), then stained by 1% alcian blue (Sigma, 05500-10G) in 3% acetic acid, followed by Mayer’s Hematoxylin and 1% Eosin Y solution. Comparisons were made among at least three independent littermates.

### Transmission electron microscopy (TEM)

Heads of E15.5 fetuses were trimmed to collect the targeted tissues, then fixed in 2.5% glutaraldehyde/0.1 M phosphate buffer (PB) for 72 h. After washing in PB overnight at 4 °C, samples were postfixed with Osmium tetroxide (OsO4) for 2 h. Samples were then dehydrated in ethanol, followed by infiltration of epon resin and propylene oxide catalyst, then embedded in epon resin. Semi-thin sections at 1 μm and toluidine blue staining were utilized to examine the samples. Ultrathin sections at 80 nm were collected and double-stained with uranyl acetate and lead citrate on carbon-coated copper grids. Sections were observed by transmission electron microscopy (Hitachi H-7100, Japan) (n = 3).

## Supplementary Information

Supplementary Information
