# Supplementary material for: Dlx5-augmentation in neural crest cells reveals early development and differentiation potential of mouse apical head mesenchyme
Source: Sci Rep. 2021 Jan 22;11:2092. doi: 10.1038/s41598-021-81434-x (PMC7822927; doi:10.1038/s41598-021-81434-x)
Supplement: Supplementary file 1 — Supplementary Information [file 41598_2021_81434_MOESM1_ESM.pdf]

Supplementary Information

Dlx5-augmentation in neural crest cells reveals early development and differentiation potential of mouse apical head mesenchyme

Tri Vu Hoang[†1], Masaki Takechi[†1], Miki Shimizu[2], Taro Kitazawa[2], Hiroki Higashiyama[2], Akiyasu Iwase[2], Hiroki Kurihara[2], Sachiko Iseki[1]*.

1. Section of Molecular Craniofacial Embryology, Graduate School of Medical and Dental Sciences, Tokyo Medical and Dental University (TMDU), Japan.
2. Department of Physiological Chemistry and Metabolism, Graduate School of Medicine, The University of Tokyo, Japan.

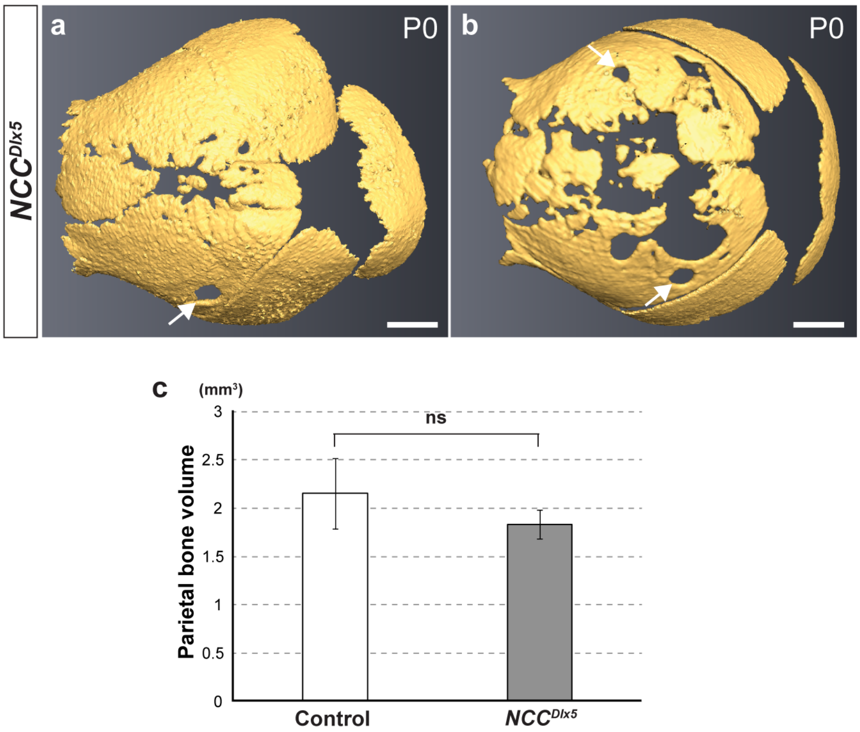

Supplementary Figure S1. Variations of heterotopic bone formation in the *NCC*^{Dlx5} calvaria. µCT images of the *NCC*^{Dlx5} calvaria at P0. Calvarium showing few minor defects in the endogenous frontal bone (**a**) and severe defects showing patchy bones (**b**), respectively. Arrows indicate bone missing areas. (**c**) Statistical analysis of parietal bone volume using µCT data; two-tailed t-test p=0.24; ns, not significant. Scale bar; 1 mm.

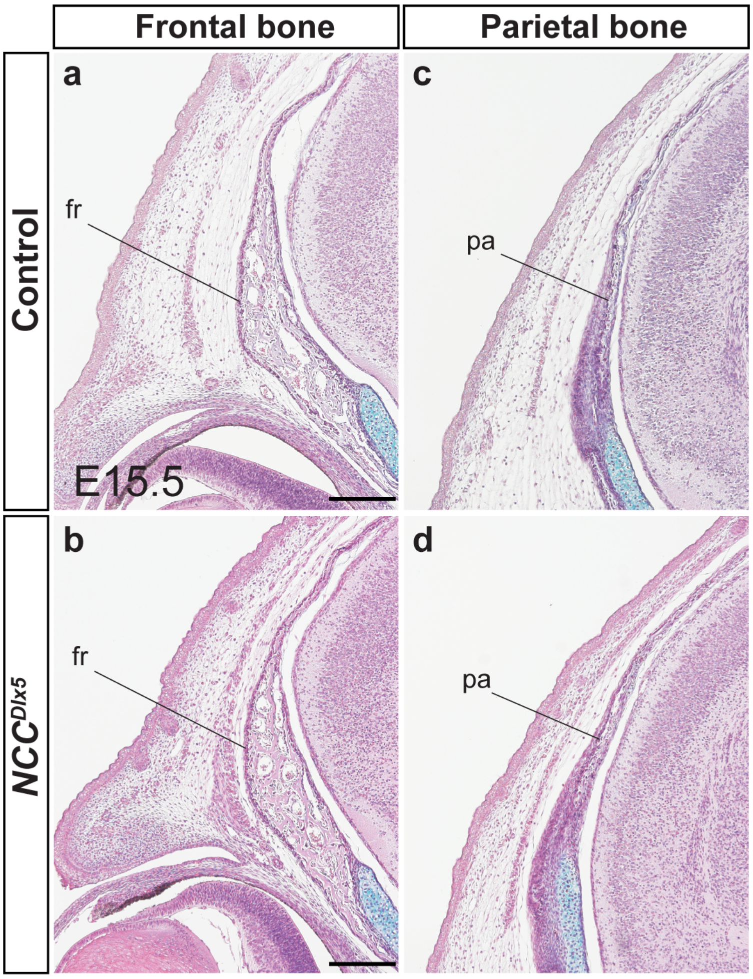

Supplementary Figure S2. Comparison of the frontal bone and parietal bone between the control and the *NCC*^{Dlx5} at E15.5. (**a**–**d**) Alcian blue and HE staining on frontal section of E15.5 control (**a**, **c**) and *NCC*^{Dlx5} (**b**, **d**) to demonstrate the frontal bone (**a**, **b**) and parietal bone (**c**, **d**). fr, frontal bone; pa, parietal bone. Scale bar; 200 μm.

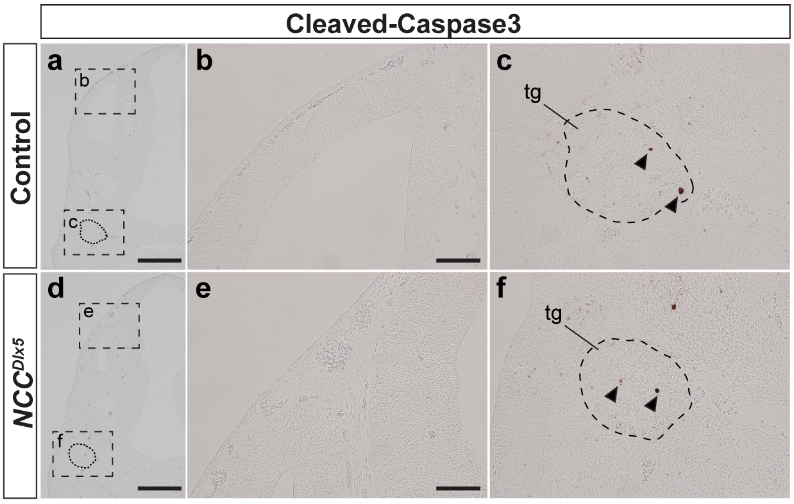

Supplementary Figure S3. Cell death analysis in the EMM at E11.5. (**a**–**f**) Immunohistochemistry staining for Cleaved-Caspase3 on frontal sections of the control (**a**–**c**) and NCC^{Dlx5} (**d**–**f**). (**b**, **c**, **e**, **f**) are high magnified images of boxes in (**a**, **d**). There is no Cleaved-caspase3 positive cell in the EMM of both control and NCC^{Dlx5} (**a**, **b**, **d**, **e**). Arrowheads in (**c**, **f**) indicate positive signals in the trigeminal ganglion. tg, trigeminal ganglion. Scale bars; 500 μm (**a**, **d**), 100 μm (**b**, **c**, **e**, **f**).

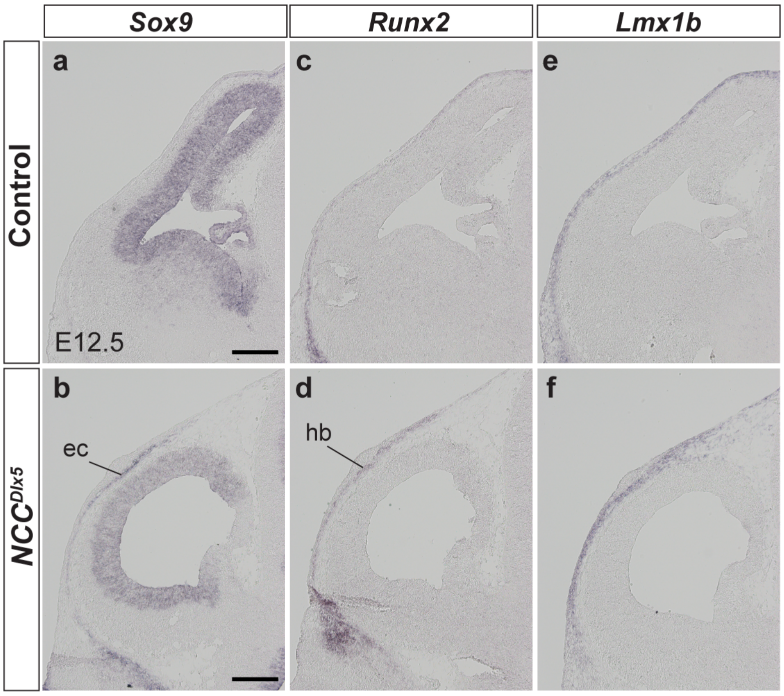

Supplementary Figure S4. *Lmx1b* is expressed in the ectopic cartilage and heterotopic bone at E12.5. *Sox9* expression (**a**, **b**), *Runx2* expression (**c**, **d**), and *Lmx1b* expression (**e**, **f**) of control (**a**, **c**, **e**) and NCC^{Dlx5} (**b**, **d**, **f**). *Sox9* and *Runx2* expressions in the NCC^{Dlx5} indicate ectopic cartilage (**b**) and heterotopic bone (**d**), respectively. *Lmx1b* expression domain includes areas of ectopic cartilage and heterotopic bone (**f**). Scale bars; 200 µm.

Supplementary Table S1. Information for riboprobes used in this study

Gene	NCBI Accession	Span	Length (bp)
Bmp2	NM_007553.3	1093..2255	1163
Dct	NM_010024.3	1178..1672	495
Dlx5	NM_010056	168..575	408
Dermo1	NM_007855.3	17..871	855
Foxc1	NM_008592.2	1913..2695	783
Lmx1b	NM_010725.3	720..1419	700
Msx1	NM_010835.2	1096..1741	646
Msx2	NM_013601.2	694..1078	385
Runx2	NM_001271631.1	2936..3832	897
Snai1	NM_011427	787..1483	697
Sox9	NM_011448	911..1728	818
Sp7/Osx	NM_130458.3	951..1626	676
